# Mechanistic Research for the Student or Educator (Part I of II) 

**DOI:** 10.3389/fphar.2022.775632

**Published:** 2022-07-01

**Authors:** Rehana K. Leak, James B. Schreiber

**Affiliations:** ^1^ Graduate School of Pharmaceutical Sciences, Duquesne University, Pittsburgh, PA, United States; ^2^ School of Nursing, Duquesne University, Pittsburgh, PA, United States

**Keywords:** mechanistic, hypothesis, physiology, biology, pharmaceutical, biomedicine, preclinical mechanistic research

## Abstract

Many discoveries in the biological sciences have emerged from observational studies, but student researchers also need to learn how to design experiments that distinguish correlation from causation. For example, identifying the physiological mechanism of action of drugs with therapeutic potential requires the establishment of causal links. Only by specifically interfering with the purported mechanisms of action of a drug can the researcher determine how the drug causes its physiological effects. Typically, pharmacological or genetic approaches are employed to modify the expression and/or activity of the biological drug target or downstream pathways, to test if the salutary properties of the drug are thereby abolished. However, experimental techniques have caveats that tend to be underappreciated, particularly for newer methods. Furthermore, statistical effects are no guarantor of their biological importance or translatability across models and species. In this two-part series, the caveats and strengths of mechanistic preclinical research are briefly described, using the intuitive example of pharmaceutical drug testing in experimental models of human diseases. Part I focuses on technical practicalities and common pitfalls of cellular and animal models designed for drug testing, and Part II describes in simple terms how to leverage a full-factorial ANOVA, to test for causality in the link between drug-induced activation (or inhibition) of a biological target and therapeutic outcomes. Upon completion of this series, students will have forehand knowledge of technical and theoretical caveats in mechanistic research, and comprehend that “a model is just a model.” These insights can help the new student appreciate the strengths and limitations of scientific research.

## Introduction

Students in STEM fields learn research methods, experimental design, and statistical analyses in the classroom but not necessarily how to interpret data or understand their meaning. Using the analogy of a child taking apart a watch and studying the individual tiny pieces, but failing in how to tell time, Bechtel argued, “the full causal account of a mechanism must include the causal processes at all levels or it will not be able to account fully for the phenomena” (Bechtel, 2010). Mechanistic reasoning and cause-*versus*-effect testing have gained considerable traction with the emergence of “hypothesis testing,” a relatively intuitive process for beginner students (Mil et al., 2016). However, hypothesis testing is an exercise fraught with caveats, which may not be appreciated until time, effort, and monies are lost. Thus, the goal of this two-part series is to highlight a few of the major caveats related to hypothesis testing and mechanistic reasoning in preclinical disease models.

## This Educational Series Does Not Replace Treatises on Experimental Design, Rigor, or Statistics

Budding researchers may not initially appreciate that results are not necessarily biologically meaningful because there is a statistically “significant” effect, or realize that published data may have no practical implications (Anderson, 2019). Conversely, a result with a *p* value slightly greater than the accepted 0.05 threshold may have important implications, and may point the researchers toward repeating the entire experiment (Berry et al., 1998). For discussions of the history behind hypothesis testing and the challenges in determining statistical significance vis-à-vis practical importance, the reader is guided to the work of statisticians (Marino, 2014; Greenland et al., 2016; Anderson, 2019; Greenland, 2019; Ioannidis, 2019; Curran-Everett, 2020; Schreiber, 2020).

For guidelines on the reporting of *in vivo* experiments on animals, the reader can consult the ARRIVE guidelines (Percie du Sert et al., 2020a; Percie du Sert et al., 2020b). The ARRIVE guidelines were introduced to the bioscience research community to improve the reproducibility and rigor of animal work, by encouraging detailed descriptions of the following: 1) experimental design, 2) sample size, 3) inclusion and exclusion criteria, 4) randomization of experimental units to minimize confounders (*e.g.,* order of treatment, animal cage location, *etc.*), 5) blinding of investigators (during group allocation, testing, or data analyses), and 6) statistical methods, including assumptions of normality and homoscedasticity, *etc*. Additional helpful guidelines on reporting biomedical research have been published by Schlenker (2016), Weissgerber et al. (2018).

It is not the goal of this brief commentary to reiterate the abovementioned, comprehensive work or to replace any texts on statistics. Rather, our foci in Part I are on a few common caveats of preclinical experimentation on pharmaceutical drug action. Also out of scope of this series is a discussion of the historical origin of mechanistic thought in physiology (*e.g.,* Claude Bernard’s work), the distinction between mechanistic, mathematical, or narrative thinking, and some of the roots of the scientific process in Hellenic and Hebraic traditions (de Leon, 2018).

## Pedagogical Gap

STEM educators are responsible for training students in the rigorous conduct and evaluation of mechanistic research. However, the PubMed search terms “(education) AND (mechanistic) AND (hypothesis)” or the alternate search terms “(teaching) AND (mechanistic) AND (hypothesis)” do not capture publications focused on teaching students how to identify mechanisms of drug action or the associated caveats. To fill this gap, we will discuss problems related to testing two commonly-encountered hypotheses, using preclinical drug testing as a teaching tool:

Hypothesis 1 The pharmaceutical drug candidate (or any kind of intervention) reduces the toxic effects of preclinical disease by upregulating the function of a protein

Hypothesis 2 The pharmaceutical drug candidate (or any kind of intervention) reduces the toxic effects of preclinical disease by downregulating the function of a protein

To test these hypotheses, it is necessary to determine if the therapeutic effects of the drug can be abolished (or at least attenuated) when the proposed mechanism of action has been interfered with. This is discussed in detail in Part II of this two-part series. In our examples, the null hypotheses state that the drug continues to mitigate the toxic effects of preclinical disease, even when the function of the protein is experimentally manipulated. However, rejection or retention of the *null* hypothesis will not be argued further in this series (Greenland, 2019). Rather, the focus is on the *test* hypothesis—as in a modern grant proposal.

The reader may wonder if the researcher really needs a mechanistic hypothesis to commence their laboratory work. The short answer is, “Of course not.” The beginner does not even need a non-mechanistic hypothesis—they can perform a study to simply observe and describe a biological phenomenon. Unlike mechanistic work, descriptive and observational studies are not dependent upon crafting any *a priori* hypothesis. Although this type of descriptive research may be viewed less favorably by reviewers of papers and grant applications, it can be less biased than hypothetico-deductive research, by virtue of its exploratory and open-ended nature (Marincola, 2007; Casadevall and Fang, 2008). The beginner may find that descriptive research is essential in the initial phases of the project, and without it, they might not be able to formulate a rational mechanistic hypothesis for follow-up work. Descriptive and hypothesis-driven research are complementary, rather than mutually exclusive, as they serve to propel each other forward (Kell and Oliver, 2004).

For drug discovery, drug development, and identification of the biological mechanism of action of a drug candidate, scientists rely upon the use of *in silico* computational methods, *in vitro* studies on cells grown as monolayers in culture dishes (including high-throughput screening work) or as three-dimensional organoids, *ex vivo* studies on organs or organ slices, and *in vivo* studies in whole animals (usually small rodents). These models share some of the flaws discussed below.

## Problem 1: Understanding the Basic Flaws of Disease Modeling

Humans and wild animals are difficult to model in the lab because they display greater genetic diversity than animal colonies at research institutions and do not live in tightly controlled temperature conditions or have *ad libitum* access to clean food and water. Inbreeding and substrain effects in animal colonies have long plagued mechanistic research (Bryant et al., 2008; Boleij et al., 2012; Zhao et al., 2019). The fallacy of “standardization” of research models (Würbel, 2000; Voelkl et al., 2018) is not widely discussed in the biological, pharmaceutical, or biomedical sciences (nor are atypical housing and nutritional conditions), but the more often a given physiological effect is observed across strains, species, and otherwise heterogeneous models, the more generalizable the effect is—and the more likely to translate to humans.

The evolutionary divergence of rodent *vs*. hominid ancestors need not stymie the study of physiological processes in the laboratory. Despite the great divergence of the telencephalon of these two groups, the common refrain that rodents are too different from humans to be useful is incorrect. Because we do not yet understand the etiology of many human diseases, it is difficult to know what to “induce” in the rodent (or even the nonhuman primate) to feasibly model the human condition. Hence, the pitfalls may lie in what we *fail* to do to cultured cells or animals to model the disease, not in lack of translation of physiological principles across species or models.

Some exceptions to the above might involve inherited or genetic conditions, as DNA sequences are relatively easy to manipulate. For example, investigators have leveraged disease-causing mutations in human induced pluripotent stem cells in the hope of avoiding the pitfalls of using nonhuman species (Chen et al., 2020; Schutgens and Clevers, 2020; Tran et al., 2020). Although studies in human induced pluripotent stem cells have the limitation of being conducted *in vitro*, cell culture affords exquisite control over the independent variables (*i.e.,* the factors that the scientist changes systematically to study their effects on the dependent variables/measurement outcomes).

Both preclinical cellular and animal models occupy essential places in mechanistic research, as they allow for the appropriate randomization of groups and manipulation of environmental and genetic independent variables, which are difficult to achieve or unethical in human subjects. Nonetheless, it is important for the student to understand that “a model is a model.” By definition, a model can never be the human disease itself, even if enthusiasm runs high when it is introduced. In sum, fully generalizable or clinically translatable observations are difficult to attain, especially when financial, technical, or human resources are limited. It is therefore prudent to adopt a skeptical but also forgiving attitude.

## Problem 2: Misapplying the Dogma of “Form Follows Function”

In both descriptive and mechanistic studies, multiple technical approaches should be used to confirm the data from different angles to ensure generalizability—including independent measurements of anatomical structure and physiological function. Although structure clearly reflects function, this doctrine can be misapplied, because technical assays are not all-encompassing and only measure *specific aspects* of structure or function. For “structural” measurements of cellular viability, cell numbers are typically counted by microscopy. For functional measurements of cellular viability, it is common to measure the energy capacity of cultured cells (Posimo et al., 2014). However, the therapeutic intervention might stimulate energy production per cell; the conventional assumption that bioenergetic supplies are in proportion to cell counts is then invalid. Conversely, cell counts might show that the drug therapy prevents cell loss *in vitro* or *in vivo*, but none of the preserved cells might be functioning properly—perhaps the cells are structurally present, but they might be incapacitated by low energy levels.

For *in vivo* studies involving drug testing, functional assessments of measurement outcomes are often used to complement the structural data. For example, a battery of behavioral tests can rule out the possibility that brain cells are structurally protected by the intervention at the histological level of analysis, but unable to fulfill their physiological roles. Aside from counting cells, scientists can also record electrophysiological properties, such as, for example, long-term potentiation as one of the bases of memory formation, using live animals or slices of the brain (Lynch, 2004).

Although many texts claim that structural changes need to be profound to be manifested as observable changes in organismal behavior, cell loss probably does not need to be dramatic to generate a clinical syndrome, as was once held (Cheng et al., 2010). In addition, neural control over behavior is not mediated by unitary brain regions as is sometimes presented, but, as argued by electrophysiologists as far back as 1970: “the available evidence indicates that the substrate for control of behavior must be located within a variety of sites in the central nervous system” (Andersen et al., 1970).

It is also underappreciated that biochemical, behavioral, and histological assays can be confounded by the stress and anxiety that the investigator provokes in the animal, an effect that may be greater for male researchers (Sorge et al., 2014). To combat this, frequent animal handling is recommended to temper activation of the hypothalamic-pituitary-adrenal axis (van Bodegom et al., 2017). Experiments should also not be performed soon after a drastic change in animal housing conditions, such as animal transportation to a new vivarium. For additional factors that can confound interpretations of animal work, please see the writings of Stacey Rizzo et al. (2018).

## Problem 3: Insensitive Measurement Tools Readily Beget False Outcomes

**FIGURE 1:**
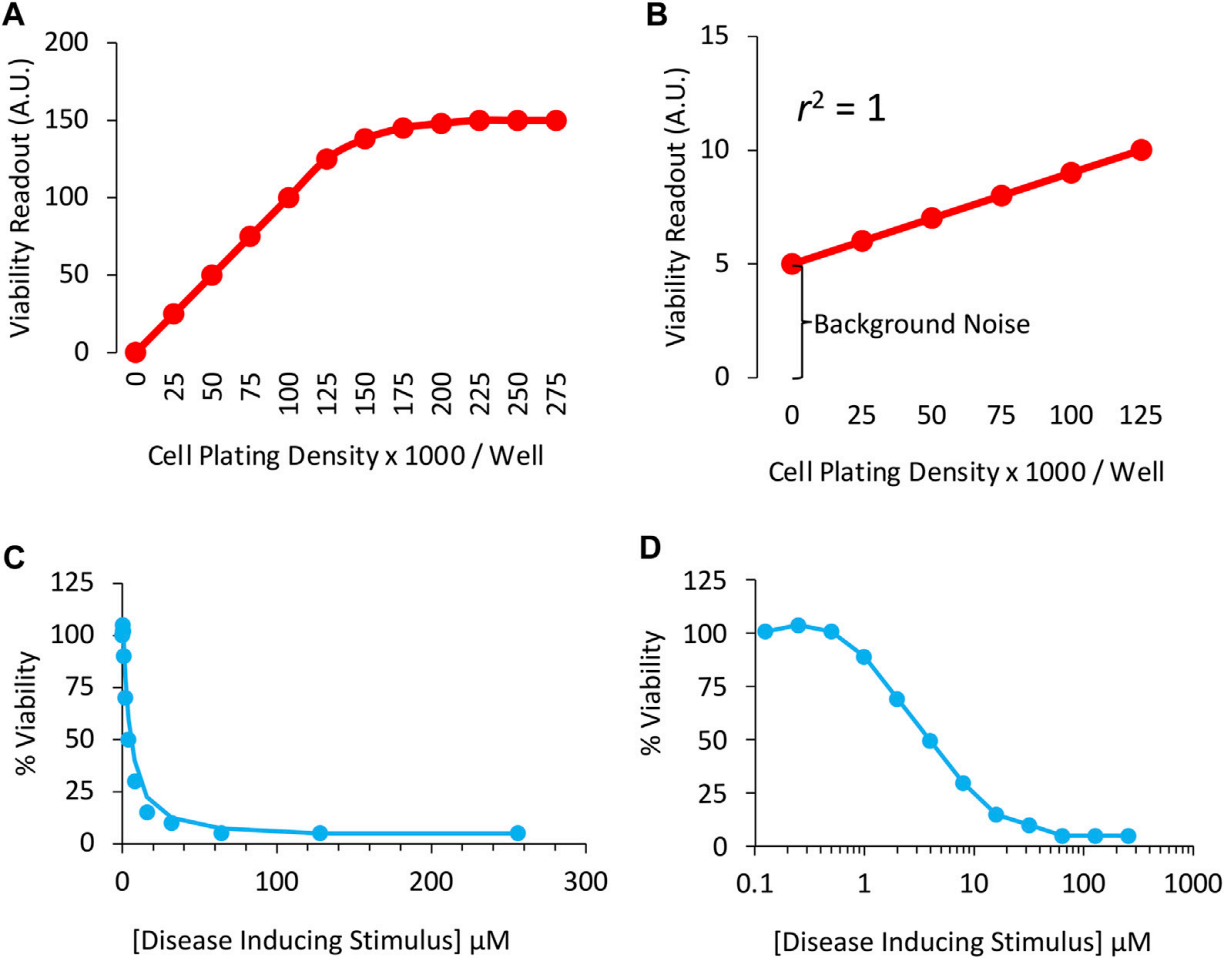
Sensitivity of the *in vitro* viability assay in cultured cells: **(A)** Correlation between cell culture plating densities (*X* axis) and cellular viability measurements (*Y* axis). Note the measurement plateau at plating densities >125,000 cells per well. The sensitivity of the assay is inadequate in this range and, therefore, the student will fail to observe any effects of treatment on viability at high cell densities. **(B)** A perfect correlation between plating densities and viability measurements is obtained, but background noise accounts for too much of the positive signal plotted on the *Y* axis. High background noise masks the size of differences across groups. **(C)** Concentration-response or concentration-effect curve for the disease-inducing stimulus in a cell culture-based viability assay, plotted on a linear scale. **(D)** Concentration *vs.* effect curve for the disease-inducing stimulus in a cell culture-based viability assay, plotted on a logarithmic scale. The LC_50_ (or IC_50_) in this example is 4 μM of the toxicity-inducing stimulus. Two-fold (rather than ten-fold) changes in concentration are employed to promote greater confidence in the LC_50_ measurements.

Assay validation is an important first step in any preclinical research, as insensitivity of measurement tools can result in Type II errors (*i.e.,* false negatives). For example, when relying on viability assays of cells plated on a dish, seeding cells in the wells of the dishes at a wide range of densities can help ensure that the assay resolves changes in cell numbers accurately (Figure 1). As an example, if we assume that the dynamic range of a particular viability assay only spans 0 to 125,000 cells per well, cell densities above 125,000 cells/well will not be resolved as a proportional change in the readout (Figure 1A). In this example, the investigator would not be able to observe effects of treatment on viability at the highest cell densities, and might report a false negative.

In Figure 1B, there is a perfect correlation between cell density and the viability readout, because the idealized data points converge upon a straight line. In real-life examples, the scatter of individual data points is higher than shown in Figures 1A,B, which would reduce the coefficient of determination (abbreviated *r* ^2^), defined as the proportion of variance in the dependent variable (the outcome measurement, plotted on *Y* axis) that is predictable from the independent variable (cell density, plotted on *X* axis).

Although the data in Figure 1B are linear and the coefficient of determination lies at the maximum of 1.0, the viability assay is too insensitive. The slope of the red line is low, and the background noise is high. As the density of the cells doubles from 25,000 to 50,000, the readout shifts from 6 to 7, a mere 17% change. Ideally, the assay readout should increase by twofold when cell numbers double.

Background values could be subtracted from the other readings in this example, because the readout in empty wells cannot be attributed to cellular material and, therefore, do not reflect viability. On the other hand, when background noise is high, it is better to identify and remove its source, such as cell culture media-coloring agents (Ettinger and Wittmann, 2014), or to find an alternative assay. In Figure 1A, the *Y*-intercept is zero when there are no cells plated in the wells, signifying lack of background noise.

## Problem 4: Undervaluing the Temporal Dimension

The importance of timing is often underestimated in biomedical research, because therapeutic interventions may only be effective in patients before irreparable damage (*e.g.*, irreversible cell loss) has taken root. On the other hand, if the intervention is tested before *any* clinical symptoms have surfaced, the translatability of the study is compromised, as patients seek treatment only once a recognizable syndrome has emerged.

Ideally, the optimal temporal windows for drug delivery and the temporal kinetics of biological responses should both be identified; if drug-induced protection is only measured at acute stages but wanes over time, then the therapeutic intervention is less clinically meaningful. Merely transient protection would not benefit the patient in the long run. For example, when modeling acute brain injuries, waiting for a minimum of 1 month post-intervention is recommended, to ensure that the therapeutic effects are not fleeting, according to RIGOR guidelines (Lapchak et al., 2013).

Aside from examining the temporal progression of the disease and testing the characteristics of drug delivery [time of initiation of treatment, frequency of delivery, circadian time of drug delivery (Esposito et al., 2020), *etc.*], the amplitude (intensity) of the experimental disease and the dose of the therapeutic drug also need to be titrated, if the lab can afford the increased cost. For example, researchers need to avoid models with intense pathology that cannot be tempered by *any* form of intervention, or else the hypothesis that the drug is protective is untestable. In sum, the timing and intensity of disease induction and therapeutic interventions should be chosen with an eye toward clinical translation, wherever technically and financially feasible.

## Problem 5: Titrating the Intensity/Dose of the Experimental Disease and the Pharmaceutical Drug Candidate

One important outcome of disease modeling may be the loss of cells from a particular organ. If the disease model is severely toxic and cell loss is massive, it will be difficult or impossible to find an effective intervention (see above section on “Undervaluing the Temporal Dimension”). Thus, it is common to titrate the intensity of the disease model, such that cell viability approximates 50% of the control values observed in control, non-diseased cells or animals.

An example of viability data designed to explore the *in vitro* toxicity of a cellular disease model is displayed using a linear scale in Figure 1C and a logarithmic scale in Figure 1D. Two-fold changes in concentration of the toxicity-inducing stimulus were applied in this example. Although ten-fold changes in dose/concentration are common in the literature, two-fold changes promote greater confidence in the LD_50_ or LC_50_ estimate, defined as the lethal dose or concentration leading to loss of ∼50% of the biological population under study, respectively. With transgenic animal models of disease, the two-fold titration approach described above is not feasible. Instead, one can test the intervention at early disease stages, before the syndrome becomes too severe.

**FIGURE 2:**
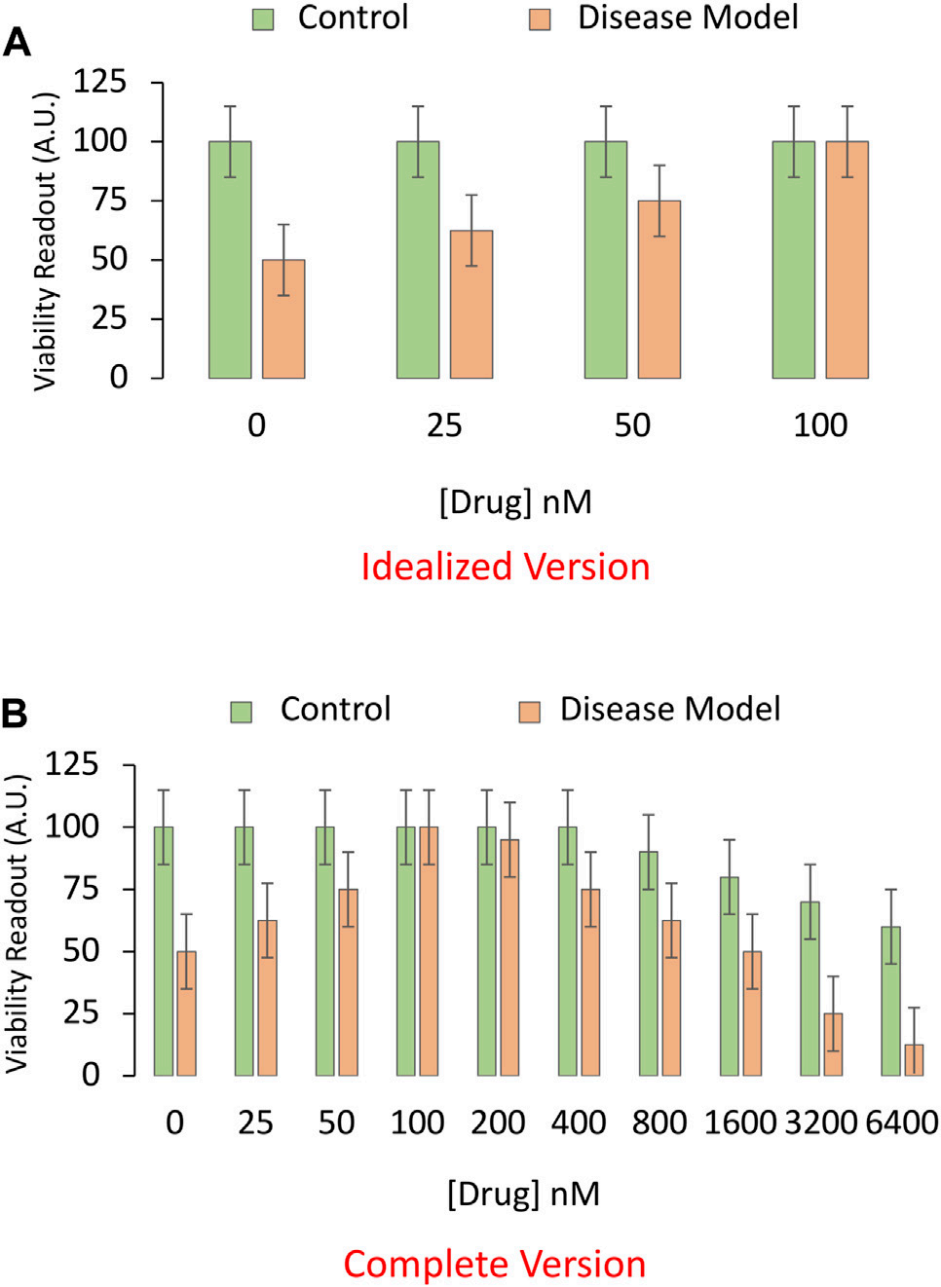
Idealized *versus* realistic versions of concentration-effect graphs for the pharmaceutical drug candidate. **(A)** A sensitive and specific viability assay is used to measure the impact of the pharmaceutical drug candidate on the survival of cultured cells in an experimental model of disease. Here, the toxicity-inducing stimulus is applied to induce the disease model at LC_50_ values (4 μM from Panels 1C,D; shown as orange bars). An equivalent volume of vehicle is applied as the negative control (shown as green bars). In this example, the drug candidate alleviates the toxicity of the preclinical disease model at relatively high concentrations, ranging from 25 to 100 nM. **(B)** A more complete concentration-effect graph reveals that higher concentrations of the drug candidate are toxic to basal viability (green bars) in cultured cells and also exacerbate the toxicity of the experimental disease (orange bars). Only the more complete version of the concentration-effect graph in panel **(B)** reveals that drug candidate has a low therapeutic index, defined as the ratio of the concentration that elicits off-target toxicity to the concentration that elicits the desired therapeutic effect.

Next, in order to find the most effective (in this example, protective) concentration or dose of the pharmaceutical drug candidate, a foreshortened concentration-effect curve is often plotted (Figure 2A), while clamping the intensity of the disease model at LD_50_ or LC_50_ values. Full dose-response or “dose vs. effect” curves are not commonly completed in preclinical animal research, often for sake of economy, and the limited range of doses is sometimes based only on previous literature or tradition. It is more common to test a wide range of concentrations in cell culture studies, but the effective concentrations in cell culture media do not always translate to the plasma or interstitial fluid of the animal, presenting another barrier to clinical translation.

In Figure 2A, the *in vitro* disease model leads to 50% loss of cellular viability in the first set of bars, and the drug prevents this toxic effect in a concentration-dependent manner, in the second to fourth set of bars. Not all drugs will display 100% efficacy in mitigating the sequelae of the toxic disease, as shown in the fourth set of bars of this idealized figure. The graph is also incomplete, because virtually all drugs—including those designated as therapies—will reduce viability if applied in sufficiently high quantities (Figure 2B), leading to an inverted U-shaped pattern, rather than the sigmoidal curves favored in textbooks. In short, the dose or concentration of the candidate drug will determine whether or not it has poisonous qualities, as noted centuries ago by the toxicologist von Hohenheim (commonly known as Paracelsus) (Leak et al., 2018).

## Problem 6: Choosing the Appropriate Controls

Assay specificity is as important as assay sensitivity and is partly addressed by the choice of controls. The inclusion of positive control groups helps to ensure that the assay can indeed resolve the expected differences. Conversely, negative control groups serve to confirm that the assay does not report differences when none are supposed to exist. In our example, the drug was applied in the presence of the disease-inducing stimulus as well as in the presence of its vehicle (the non-diseased negative control). Often, the control or *in vitro* vehicle of choice is dimethyl sulfoxide, due to its miscible properties. As stated above, “the dose makes the poison,” and dimethyl sulfoxide can be toxic above a surprisingly low threshold (Galvao et al., 2014). It is, therefore, sometimes necessary to include an “untreated” control, in addition to dimethyl sulfoxide.

All experimental groups should receive the same final volume and concentration of vehicle. If the group treated with the highest concentration of a drug also receives the greatest amount of vehicle, but the controls fail to account for this, the researcher might erroneously conclude that the effects are due to the drug, when they are actually attributable to a higher volume of vehicle than in the “control” group.

**FIGURE 3:**
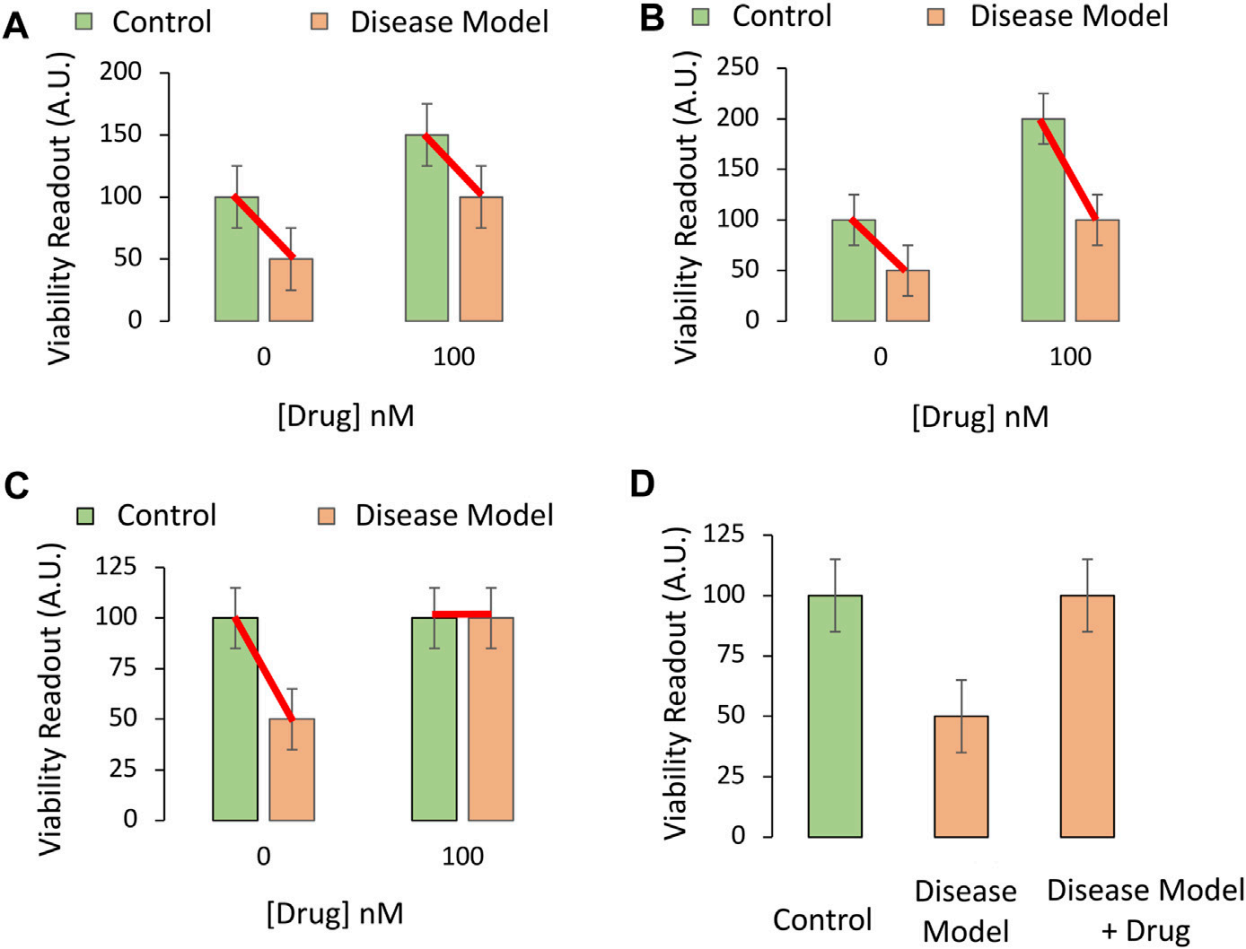
Testing if the pharmaceutical drug candidate modifies the impact of the experimental disease on cellular viability *in vitro*. **(A,B)** The drug candidate raises the viability of cultured cells at the indicated concentration of 100 nM (chosen from Panels 2A,B), regardless of experimental disease (orange bars) or control (green bars) conditions. The drug only raises baseline viability; it does not truly protect cells against the loss of viability under disease conditions, even if the two orange bars are significantly different in *post hoc* pairwise comparison tests. **(C)** The drug completely prevents the loss of cellular viability with disease at the indicated concentration. The impact of the experimental disease on cellular viability is therefore modified by the drug candidate. **(D)** If one of the control groups is not included [second green bar from panel **(B)** is missing], the data might appear as shown in this panel. This drug candidate may fail to modify the toxic impact of disease, but that would not evident from these three groups alone. If the primary readout involves pathological markers that are truly absent (zero) in the non-diseased control group, then the experimental design in panel **(D)** and a one-way ANOVA might be sufficient to test the hypothesis.

The importance of administering the pharmaceutical drug candidate in both the absence and presence of the disease model is displayed in Figure 3A. If the drug increases viability in cell culture under baseline conditions, even in non-diseased control cells, and it increases cell viability to a similar degree under disease conditions, then the conclusion that “the drug changes the impact of disease” is false. This conundrum is addressed by choosing the proper test, a two-way ANOVA on Gaussian and homoscedastic data, as per the assumptions of this parametric test.

The two-way ANOVA is used to assess the impact of two independent variables (factors) on a dependent variable (outcome measurements), as well as any statistical interaction between the two independent variables. In this simplified, cell culture-based example, the first factor is “disease state,” of which there are two levels: i) Administration of vehicle (e.g., saline) to the non-diseased control group, or ii) delivery of the toxicant stimulus that induces the disease model at LC_50_ values. This factor is plotted as green *versus* orange bars in Figure 3.

The second factor is “treatment,” of which there are also two levels: i) Vehicle or ii) the pharmaceutical drug candidate that is being tested for its protective properties. For simplicity, only one concentration of the latter is displayed in Figure 3 and plotted on the *X* axis.

Factors should not be confused with *levels*. If more concentrations of the drug or the disease stimulus were added, a two-way ANOVA would nevertheless be employed, as the influence of only two factors—disease state and treatment—is still being tested.

The dependent variable in Figure 3 is the preclinical outcome (*in vitro* cellular viability). In Figure 3A, there is no statistical interaction between the drug and disease when cellular viability is measured. In simpler words, the drug improves cellular viability by the same degree, *whether or not the cells are diseased*. When the data are graphed as red lines connecting the means, they lie in parallel, because the two independent variables do not influence each other’s impact on viability (Figure 3A).

In Figure 3B, the red connecting lines are not exactly parallel, but in neither Figure 3A nor Figure 3B can the investigator conclude that the drug is protective against disease. Even though there is a statistically significant interaction between the two variables in Figure 3B, the loss of viability with disease is still 50% of control, non-diseased values, in both the absence and presence of the drug.

Figure 3C reveals a protective effect of the drug and an interaction between the two variables. Even if the protective efficacy of the drug was not as high as in idealized Figure 3C, but reproducible, the drug might show promise in the battle against preclinical disease.

A word of caution—a simple pairwise comparison test after the ANOVA will not account for the change in baseline viability in the non-diseased group. Rather, the *post hoc* test might reveal a statistically significant difference between the two groups colored orange in Figures 3A,B, whether or not the drug is truly protective.

Finally, beginners may fail to include the non-diseased control group treated with the therapeutic drug candidate (second green bar, Figures 3A–C) and perform a one-way ANOVA on three groups only, as in Figure 3D. The latter experimental design is sometimes justified, but it will fail to reveal if the increase in viability with the drug is due to an increase in *basal* viability, or is attributable to actual modification of disease processes. Only the full-factorial, two-way ANOVA can reveal whether the drug modifies the impact of the disease in this example.

For a more extended discussion of ANOVAs, please refer to the classic descriptions by Ferguson and Takane (1989) or more recent work by Schreiber. For discussion of the importance of reporting statistical interactions, the reader should also consult the writings of Garcia-Sifuentes and Maney (2021).

## Problem 7: Identifying a Suitable Biological Target

The potential biological targets of a rationally designed drug may first be identified with computational tools (*e.g.*, molecular modeling) and then biologically validated at the bench (Silakari et al., 2021). The modeling of the interaction between a drug and its target is based on the three-dimensional structure of each molecule, which is represented in a mathematical fashion by leveraging the equations of quantum and classical physics. Computer simulations can help predict the thermodynamic and physicochemical properties of the drug-target interaction, usually at an atomistic level. Molecular modeling can be an economical alternative to using a series of binding assays to find a membrane receptor target out of dozens of possibilities. Thus, molecular modeling is useful to narrow down the list of possible targets engaged by the drug.

Even when testing a relatively new drug, there is not usually a complete vacuum of knowledge about potential biological effects. Besides molecular modeling, one way to steer the wet bench work is to read the literature and determine if there exist any data linking the candidate drug—or similar classes of drugs—with the protein in question, or with homologous proteins. Even if the drug was never tested in, for example, the brain, it may have been studied in models of breast cancer. The latter knowledge is then easy to leverage in brain tissue or neural cells as a basis for repurposing the drug. If the candidate drug is entirely new, RNA sequencing or proteomic analysis can be used to identify the biological pathways that are directly or indirectly engaged by the drug, including at the single-cell level with the use of bioinformatics (Haque et al., 2017; Stark et al., 2019). These collective methods will help the student sketch the biological pathways that are directly and indirectly engaged by the drug, including those pathways downstream of receptor binding, as discussed in Part II. The proteomic analyses will shed light on the post-translational modifications elicited by drug application, and the transcriptomic analyses will shed light on gene expression changes.

While characterizing the pharmacological impact of the drug, the timing of the assay is critical (see section on Undervaluing the Temporal Dimension). If receptor activation, protein cleavage, or protein phosphorylation is hypothesized as the mechanism of action, to name a few, measurements must be made close in time to application of the drug. If changes in gene expression are expected, the cells must be allowed sufficient time to engage the transcription and translation machinery. Either way, the protein will usually need to be assessed at multiple time points. Furthermore, a vehicle control may need to be included for every time point, if the vehicle exerts time-dependent effects of its own. Students may only include a “0 h” time point as a substitute for a vehicle control, but this will not capture potential time-dependent changes induced by the vehicle itself. For example, vehicle administration *in vivo* may activate the stress axis in animals, and the investigator would ideally need to control for this variable, in a manner that a “0 h” control time point cannot.

While searching for a candidate protein that is affected by the drug, it is important to distinguish between protein “induction” and “activation,” because proteins can be activated without induction and *vice versa*. Protein induction is examined by measuring levels of the mRNA. However, cells might increase mRNA levels without any change in protein expression—if the protein is also degraded more rapidly. Thus, protein concentrations do not necessarily parallel mRNA levels, and, for this reason, it is insufficient to show that mRNA levels are affected by the drug.

In the two hypotheses mentioned above, the *function* of the protein is up or downregulated by the drug. When the overall expression of a protein is increased, there is no guarantee that it is also more active or that its function is enhanced. As an example, a pharmaceutical drug candidate might indirectly promote dephosphorylation-mediated inactivation of the protein, which may subsequently result in a compensatory increase in total protein levels, but without a net increase in the numbers of *functional* protein molecules. If the student was unaware of the dephosphorylation-mediated inactivation of the protein, as well as the compensatory nature of the delayed increase in total protein expression, they might assume that the drug increases the function of the protein and downstream pathways, when the opposite is true. For these reasons, the student is encouraged to use a number of technical tools to assess upregulation or downregulation of the protein from multiple angles.

## Conclusion

To summarize, the student is encouraged to titrate the amplitude of the experimental disease and identify the optimal dose of the drug candidate prior to combining these two factors in a full-factorial experiment. It is helpful to include sensitive and specific functional assays of the protein target across time after drug administration, as well as negative and positive control groups. In Part I of this series, students have learned how to avoid some of the pitfalls common in biomedical research. In Part II of this series, the reader will learn how to test the hypothesis that a particular molecular target of the drug (a protein) mediates its protective effects, while avoiding common errors in data interpretation. To link to Part II, please see this hyperlink https://www.frontiersin.org/articles/10.3389/fphar.2022.741492/abstract.

## Data Availability

The original contributions presented in the study are included in the article/supplementary material, further inquiries can be directed to the corresponding author.
